# Dr. Kinglake, on Phthisis Pulmonalis

**Published:** 1809-03

**Authors:** Robert Kinglake

**Affiliations:** Taunton


### *To the Editors of the Medical and Physical Journal.*

GENTLEMEN,

THE last stage of Phthisis Pulmonalis often presents a most noisome and afflicting scene of disease; but the familiarity of the occurrence, and the habit of observing the morbid appearances of that forlorn state, have induced a disregard to any possible mischief that may arise from an unwary and unduly protracted attendance on those occasions.

It is well known that diseases of various descriptions generate and evolve during their existence, and more particularly towards their termination, a certain property, whether of a *material* or *motive* nature, that has a tendency to impart its morbid quality to those who may come within the sphere of its active power, and whose constitutional susceptibility may be favourable to its infectious influence. The occasional extension of common fever by personal intercourse, as well as that of a more typhoid character, evinces its capability of communicating infection. This effect, however, is not dependent on the mere existence of an infectious power, and on an opportunity of imparting that infection, but on a variety of collateral circumstances with which the relative efficiency of these causes is essentially connected. These circumstances are chiefly a given concentration of the infectious cause, and a certain capability or aptitude for receiving its active impression; if either the one or the other should fail, happily no effect is produced. This incongruity fortunately often prevents and confines the empire of infectious disease within comparatively narrow limits.

Matter, in all its various arrangements, possesses properties characteristic of the particular nature of its existence. Its active powers in some forms are more direct and efficient than in others; but in no shape whatever is it reasonable to suppose it quite inert. In the peculiar conditions necessary to animal health, no deviation can happen consistently with the avoidance of disease. When disease actually occurs, effects, different from those of health, necessarily arise. These will be various, according to the degree or intensity of the morbific cause, and will possess properties and exert an influence correspondent to the particular nature of the active power. It is in this mode of estimating the efficiency of morbid power, that some just conclusion may be drawn respecting the infectious agency of diseases in general, and more especially of those that seem directly to impart their morbid influence in suitable circumstances for its operation.

In the advanced stage of phithisis pulmonalis, more particularly, occurs an aggravated state of disease that would seem well adapted to concentrate and give full effect to any infectious powers, which, in common with all diseased changes from the healthful condition of life, it may be supposed to possess. The hectic commotion, the wearing decomposition, the consequent extrication of the constituent principles of animal matter, and the new arrangement which may be formed of these substances, saying nothing of the peculiar morbid action obtaining in this disease, and which may be embodied and transferred, render it extremely probable that this disease is capable of exerting infectious influence, and that it actually does appear to me verified by some instances of its having been imparted to persons closely attending the last stage of its existence. I have more particularly in my recollection the case of one sister sleeping with and closely associating with another, who laboured under phthisis pulmonalis in its worst form, in whom, ulceration of the lungs appeared to have taken place, accompanied with the most embarrassing state of cough, fœtid expectoration, and cadaverously smelling night sweats. Death, at length, closed this hopeless scene of disease. The surviving sister, who had anxiously watched and assisted during its destructive progress, soon became disordered by strictured breathing, painful cough, febrile rigors and heats, loss of appetite, and sleepless nights; to these symptoms were speedily added, bloody expectoration, night sweats, and every other appearance of an advanced and irreparable state of pulmonary consumption, which soon terminated as the preceding instance.

It should be remarked, that in neither of these cases did hereditary or scrophulous predisposition to pulmonary disease appear to exist. Both sisters had been uniformly free from every suspicion of even a tendency to the disease, until it actually surprised the one, and overtook the other, as a probable consequence of an unremitted personal intercourse. I have often had an opportunity of observing a threatening degree of pulmonary affection to have apparently resulted from incautiously inhaling the distempered vapour of phthisical patients, but do not recollect any any instance in which the effect was so direct and unequivocal as in the case recited.

It does not follow, that on every occasion in which injury is done, that it should be of the irreparable nature which marks pulmonary consumption; it may disorder the lungs without inducing that deeply diseased affection of them; it may produce a disposition to asthma, and an aptitude for pulmonary ailment on occasions which might not otherwise have become active. In this view of the subject, it would be adviseable to avoid being too closely inmated with patients in the dying stage of pulmonary disease. At a period when no benefit can be rendered and much mischief may arise, it will be no imputation on humanity to withhold unnecessary attendance.

It does not appear that the ground for suspecting the infectious nature of phthisis pulmonalis would warrant a belief that it is of that active quality as would justify even the timid in not paying a due degree of personal attention to the diseased; it is the incaution of dwelling with such patients, in leaning over them by the hour, in breathing in the immediate atmosphere of their lungs without restraint, that deserves to be pointed out as exceptionable, because in itself useless, and certainly fraught with serious evil to those who may pursue that hitherto unobjected and supposed unobjectionable course of conduct.

The infectious nature of diseases is but inadequately understood, the circumstances under which it obtains have not been traced with sufficient distinctness. Some forms of disease, such as the small-pox, measles, syphilis, &c. have attained to a precision of power and clearness of character that have familiarized their infectious quality to common observation; yet this obvious fact has not been analogically extended either to the diseased state in general, or to any of the modifications under which it might appear. Had this been more assiduously done than has been the case, much valuable information would have been ere now obtained on both the source and agency of morbid infection. If diseased action of a definite character should be capable of generating a certain arrangement of matter capable of imparting the specific disease to an uninfected person, as in the small-pox, measles, syphilis, &c. who is competent to say how far the process is not imitated in other instances of disease? Happily for mankind it does not often go to the full formation of the infectious influence, yet it may occasionally do it, and that it actually does so, is exceedingly likely from the examples in which diseases some-

 times times pass through families, and pervade even the inhabitants of whole districts of country, in a way that could not happen but by infectious influence.

The question which has so long agitated and divided the schools of medicine, respecting the material or motive source of disease, seems to have been very generally decided in favour of the motive doctrine. All motion, in physics at least, must originate from matter, or which is tantamount, motive power must be materially embodied, without which, it could not be contemplated as a cognisable entity. Morbid motion, therefore, when thus generated, is capable of transferring and distributing itself through the animal system. When disease arises from a point, as that induced by inoculated small-pox, the matter inserted is not diffused over the system, but the action only which the presence of that matter on the part has excited. The pustular inflammation resulting from the insertion of variolous matter is the focus of morbid action, from whence it spreads through the system; and where it induces suppuration, a source of transferable infection is furnished.

The matter thus produced, contains in its nature the property of exciting in susceptible circumstances for its influence, the same kind of action that would in turn generate more matter of exactly similar quality. All diseases, indeed, have not the power of so arranging and combining animal matter as to afford an agent so specifically infectious, as occurs in variolous, morbillous, syphilitic, and other distempers, yet it is common to all diseases to produce either material or motive powers capable of exciting morbid effects in suitable circumstances for their action, precisely analogous to those from which they respectively originated. The possibility of diseased impressions being derivable from the influence of diseases in general, with the evidence of morbid infection being actually imparted by certain diseases, and the great probability of there being a communicable quality in phthisis pulmonalis and in other disorders not hitherto considered as infectious, deserve to be regarded as a subject of high practical importance.

The fact that pulmonary consumption is an increasing disease, is at once notorious and alarming. The prevailing modes of living, may, in some measure, have contributed to its augmented frequency; but it may also be reasonably supposed, that the ordinary personal assiduities employed, in endeavouring to alleviate the distress of those who are afflicted with this ailment, may have extended its baneful dominion. Much avoidable inconvenience and risk may be be salutarily shunned in attending patients in the usually hopeless circumstances of the last stage of phthisis pulmonalis, without withholding the smallest portion of necessary aid. It is the incaution arising from an unconsciousness of possible injury from an unremitted personal intercourse on these occasions, that may prove hurtful, the requisite attendance, as in most other diseases, may be given with sufficient safety.

The supposition that an infectious quality exists in these states of disease, would likewise suggest the propriety of attempting to obviate the force of its power, by free ventilation, attentive cleanliness, and even by neutralising it, if possible, by chemical combination. The muriatic acid gas appears to combine with and to destroy the active powers of the infectious vapour generated in typhoid fevers; that, or some more appropriate chemical agent may be equally effectual in annulling the infectious quality arising in phthisical affections of the lungs. At all events it promises to be advantageous to sound an alarm of possible danger in the recited circumstances, and to require that it should be resisted by all suitable means of counteraction.

I am, &c.

ROBERT KINGLAKE.

*Taunton, Dec.* 28, 180 .

P. S. In farther support of the opinion held in the preceding communication, may be offered the testimony of a gentleman of about 40 years of age, who has just consulted me in his own case of an advanced stage of pulmonary consumption, which he (without the slightest intimation on my part) believes to have arisen from a close personal attendance on his late wife, who died a few months since of phthisis pulmonalis. He describes the fœtor of her breath to have sensibly disordered his respiration, and affirms, that he now discovers in his own breathing, an odour exactly resembling that which he had perceived in his wife's case, and the noisomeness of which he had perseveringly and fearlessly endured.

This case is certainly in point, and goes to shew, that deleterious consequences may result from indefinitely breathing either in the immediate atmosphere of distempered lungs, or in the vapour exhaling from diseased surfaces in any other part of the animal body.

